# Exploring sustainable management by using green nano-silver to combat three post-harvest pathogenic fungi in crops

**DOI:** 10.1186/s11671-024-03986-x

**Published:** 2024-03-19

**Authors:** Sudhir S. Shende, Aniket K. Gade, Tatiana M. Minkina, Pramod U. Ingle, Vishnu D. Rajput, Svetlana N. Sushkova, Saglara S. Mandzhieva, Mahendra Rai, Ming H. Wong

**Affiliations:** 1 Academy of Biology and Biotechnology, Southern Federal University, Rostov-on-Don, Russia; 2 Nanobiotechnology Laboratory, Department of Biotechnology, Sant Gadge Baba Amravati University, Amravati, MS India; 3 Department of Biological Sciences and Biotechnology, Institute of Chemical Technology, Mumbai, India; 4 Department of Microbiology, Nicolaus Copernicus University, Torun, Poland; 5 Department of Chemistry, Federal University of Piaui (UFPI), Teresina, Brazil; 6 Consortium On Health, Environment, Education, and Research (CHEER), and Department of Science and Environmental Studies, The Education University of Hong Kong, Tai Po, Hong Kong China

**Keywords:** Agriculture, Cm-AgNPs, Crop protection, Fabrication, Sustainable, Zero hunger

## Abstract

Global crop protection and food security have become critical issues to achieve the ‘Zero Hunger’ goal in recent years, as significant crop damage is primarily caused by biotic factors. Applying nanoparticles in agriculture could enhance crop yield. Nano-silver, or AgNPs, have colossal importance in many fields like biomedical, agriculture, and the environment due to their antimicrobial potential. In this context, nano-silver was fabricated by *Citrus medica* L. (Cm) fruit juice, detected visually and by UV–Vis spectrophotometric analysis. Further, AgNPs were characterized by advanced techniques. UV–Vis spectroscopic analysis revealed absorbance spectra at around 487 nm. The zeta potential measurement value was noted as -23.7 mV. Spectral analysis by FT-IR proved the capping of the acidic groups. In contrast, the XRD analysis showed the Miller indices like the face-centered cubic (*fcc*) crystalline structure. NTA revealed a mean size of 35 nm for nano-silver with a 2.4 × 10^8^ particles mL^−1^ concentration. TEM analysis demonstrated spherical Cm-AgNPs with 20–30 nm sizes. The focus of this research was to evaluate the antifungal activity of biogenic AgNPs against post-harvest pathogenic fungi, including *Aspergillus niger*,* A. flavus*, and *Alternaria alternata.* The Cm-AgNPs showed significant antifungal activity in the order of *A. niger* > *A. flavus* > *A. alternata.* The biogenic Cm-AgNPs can be used for the inhibition of toxigenic fungi.

## Introduction

Agriculture is the global economy’s resilience, and most of the globe’s population depends on it for a living. The United Nations aims for “#Zero Hunger” as an important part of their Sustainable Development Goals (SDGs). It is projected that about one-third of the overall yield is abandoned every year because of plant pathogens and insect damage. Likewise, viral, bacterial, and fungal diseases are responsible for significant yield loss [14, 15, 32, 59].

The remarkable antimicrobial capability of nanoparticles against plant pathogens has been extensively researched to establish their applications in agriculture [8, 26, 58]. Medicinal plants have many valuable bioactive compounds, including alkaloids, tannins, and phenolics. These biologically active compounds may enhance the metal ions’ transformation into biogenically functional nanoparticles in an environment-friendly standard biosynthesis route [37]. Plant components like leaves, stems, fruit, flowers, seeds, bark, roots, rhizomes, and fruit peels are used to fabricate several types of nanoparticles [10, 33, 34, 38, 43].

The present study used *Citrus medica* (L.) fruit juice to synthesize silver nanoparticles (AgNPs). The citron fruits treat malaria, cough, and cold, and the stem is used as an antipyretic. Previously, it was documented that the fabrication of copper nanoparticles by citron fruit juice demonstrated antimicrobial activity against some common bacteria: *Escherichia coli*, *Klebsiella pneumoniae*, *Propionibacterium acnes*, *Pseudomonas aeruginosa*, *Salmonella typhi*, and fungi: *Fusarium culmorum*, *F. graminearum*, and *F. oxysporum* [52].

AgNPs have been designated as ‘dynamic’ on account of their capability to employ tremendous possibilities for biological applications, particularly antibacterial, antiviral, antifungal, and anticandidal, activities even at lower concentrations [3, 24, 25, 36, 39, 45, 54]. It is a non-hazardous inorganic antimicrobial agent that efficiently eliminates roughly 650 distinct pathogenic microbes [2, 11, 43]. As for AgNPs’ usage in agriculture, researchers in this field are interested in the green fabrication method of these nanoparticles. It has already shown a promising and environmentally friendly approach [1, 16, 23, 28, 31, 48, 51, 54]. The synthesis of green AgNPs employing biogenic sources as reducing agents has been comprehensively executed. It is less expensive, and the raw materials are readily available without chemical agents that may cause toxicity to nature and humans [41]. The green synthesis method makes the large-scale production of nanoparticles lucrative and safer [37, 53, 55].

There is a pressing need to develop a unique fungicidal agent that is both safe and economical. While a study has documented the fabrication of AgNPs employing the leaf extract of *Citrus medica* (L.) and highlighted their antioxidant activity [49], the synthesis of AgNPs by the fruit juice of *C. medica* remains unexplored. To address this gap, we utilized citron fruit juice in a concentrated silver nitrate (AgNO_3_) solution to synthesize AgNPs by reducing silver ions. Additionally, we evaluated the biogenic AgNPs against three crop pathogenic fungi for their management. Thus, the current study represents a novel and timely contribution to the management of crop pathogenic fungi.

Various advanced nanoparticle characterization techniques were used for the characterization of biofabricated Cm-AgNPs, and the antifungal activity was evaluated against the pathogenic fungi causing post-harvest diseases in crops including *Aspergillus flavus*, *A. niger*, and *Alternaria alternata* for the sustainable employment of AgNPs to benefit society by decreasing yield loss.

## Materials and methods

### Biofabrication of silver nanoparticles (Cm-AgNPs)

*Citrus medica* (L.) fruits, i.e., citrons, were collected from the backyard of the Biotechnology Department, Sant Gadge Baba Amravati University, Amravati, Maharashtra, India. The acidic citron fruit juice was extracted at room temperature by crushing the pre-washed, ripening fruits and filtering through with cheesecloth and filter paper to remove the debris. The known quantity of silver nitrate (AgNO_3_) (100 mM stock made in sterile purified water) solution was put into the filtered citron juice, making its final concentration 1 mM. The resultant solution was thoroughly mixed and kept as it was until the color change was visible in the solution mix.

### Characterization

Following that, biofabricated Cm-AgNPs were detected and characterized using several nanotechnological techniques that are described in detail as below.

#### Visual detection and UV–Vis spectroscopic analysis

The direct detection of Cm-AgNPs was performed visually by observing a color change in the precursor solution. A UV–Vis spectrophotometer (Shimadzu UV-1700, Japan) was used to determine the absorbance of the Cm-AgNPs colloidal system. The diluted colloidal suspension of NPs was scanned in the range of 200–800 nm at 1 nm resolution. The NPs demonstrate strong optical density in the visible range because of the Surface Plasmon Resonance (SPR) phenomenon [42].

#### Zeta potential measurement

Using the Zeta Sizer 90 (Malvern ZS-90, USA), zeta potential measurement, i.e., the stability of Cm-AgNPs, was determined. To make the sample, 2 mL of distilled water was mixed with 30 µl of colloidal suspension. Nearly 1 mL of the sample was brought into a zeta dip cell and put in the sample cavity to measure zeta potential [27, 53].

#### Fourier transform infrared (FT-IR) spectral analysis

The FT-IR spectral analysis was performed for Cm-AgNPs on Bruker’s Alpha T FT-IR instrument, Bruker Optik GmbH, Germany, equipped with a DTGS detector working at RT and supported by the OPUS software. Using dry potassium bromide (KBr) pellets that were scanned in the range of 400–4000 cm^−1^ at a transmission mode with resolutions of 4 cm^−1^ for each sample gives an idea about the functional groups from the citron juice extract associated with the reducing, capping, and stability of Cm-AgNPs by generating the transmittance peaks that were the mean of 100 scans taken on the attenuated total reflection (ATR) diamond crystal of the FT-IR instrument [53].

#### X-ray diffraction (XRD) studies

The fabrication of Cm-AgNPs, along with the crystal structure and size, was approved by the XRD pattern. The XRD pattern of Cm-AgNPs was documented employing the Rigaku Mini Flex II Desktop X-ray diffractometer instrument and CuKα radiation (0.15418 nm). The powder of Cm-AgNPs for XRD analysis was prepared. The sample was scanned in the range of 20˚-80˚ of 2θ with a 46-s counting time. The ISDD standard software, Joint Committee on Powder Diffraction (JCPDS, Standard), made an XRD data study [42].

#### Nanoparticle tracking and analysis

The Nanoparticle Tracking and Analysis (NTA) system (Nano Sight LM 20, UK) was used to determine the size of Cm-AgNPs by subjecting diluted aliquots of the sample (5 µL of colloidal Cm-AgNPs in 2 mL distilled water) and observing the particle movement through a camera attached to the device [27].

#### Transmission electron micrograph (TEM) and selected area electron diffraction (SAED) pattern studies

For TEM and SAED analyses of Cm-AgNPs, the sample was loaded on the standard carbon-coated copper grid (400 meshes, Plano Gmbh, Germany) and subjected to IR light for solvent dry-up. A transmission electron microscope (Philips, model CM 200) functioning at an accelerating 200 kV voltage was used for the analysis. The crystalline nature and shape of Cm-AgNPs were determined and established by TEM and SAED pattern analysis [12, 53].

### Antifungal activity assessment of biofabricated Cm-AgNPs

The antifungal activity was assessed by the disk diffusion method [9] against three common crop pathogenic fungi, viz., *Aspergillus flavus* (MTCC 277), *Aspergillus niger* (MTCC 4325), and *Alternaria alternata* (MTCC 7202). The suspension of a fully grown culture of the test fungi was made and streaked onto the Potato Dextrose Agar (PDA) plate. There were four treatment disks: Cm-AgNPs alone (20 µL/disk), Cm-AgNPs in combination with the standard antifungal agent Ketoconazole (KT) (1 µg/disk), plant extract as a negative control, and KT alone as a positive control (1 µg/disk) were placed onto the agar surface and incubated for 4 days at 25 ± 2 °C. Subsequently, after the end of the incubation period, the diameter of the inhibition zones (ZOI) was measured using the zone measurement scale (Hi-Media, Mumbai, Maharashtra, India). The average ZOI was calculated for each treatment, as the experiment was conducted in triplicate. The graph was plotted against test organisms for treatments *vs.* ZOI (diameter in mm) with their standard deviation (SD).

## Results

### Synthesis and characterization of biofabricated Cm-AgNPs

**Fig. 1:**
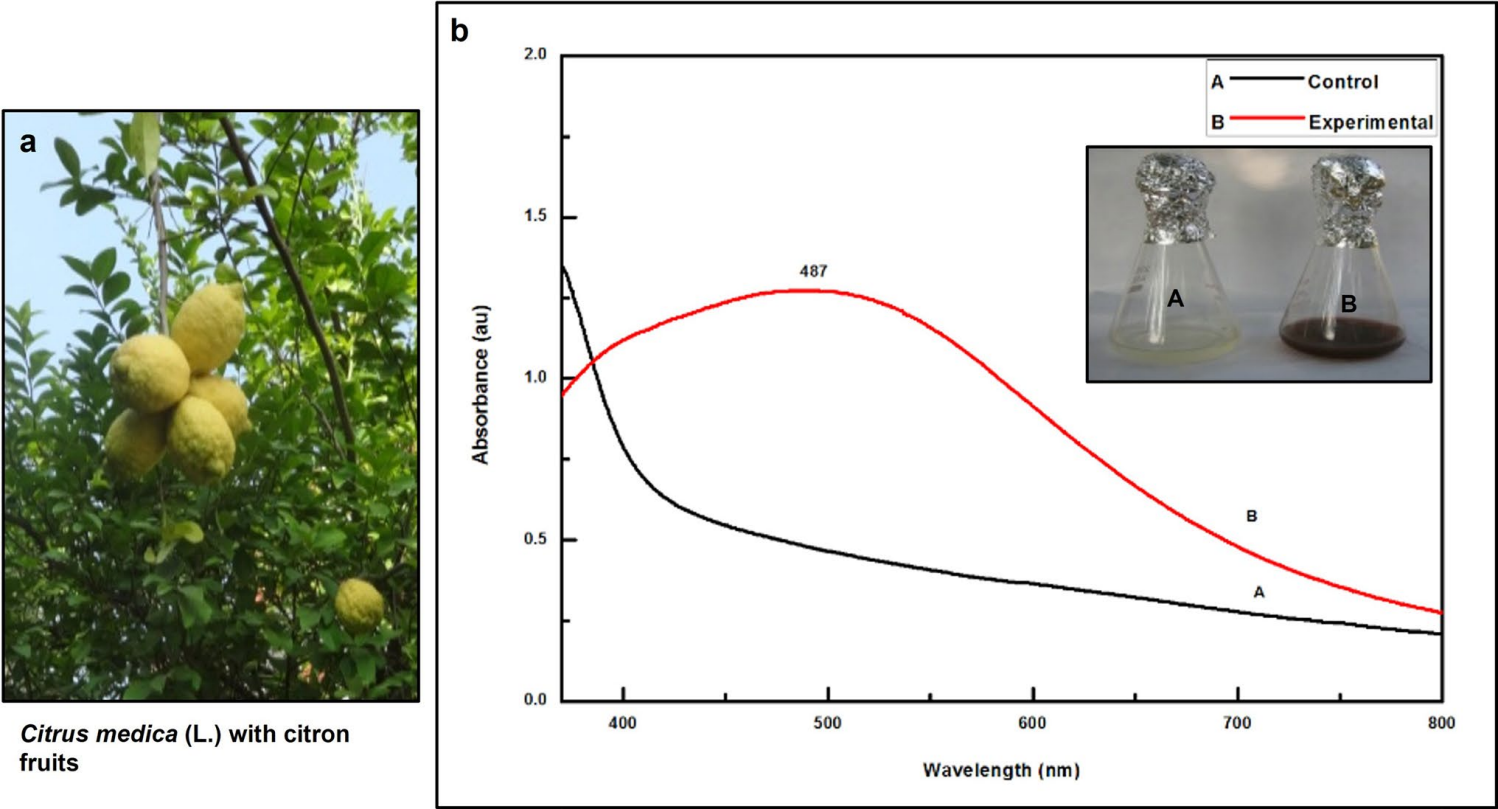
**a** Biofabrication of AgNPs using *Citrus medica* L. fruit juice **a** Photo of citron fruits; **b** UV–Visible spectral analysis for Cm-AgNPs **A** Control (Citron juice); **B** Experimental Cm-AgNPs [inset figure **A** Control (Citron juice), **B** color change of precursor solution after treatment with 1 mM AgNO_3_

The biofabrication of AgNPs using acidic *C. medica* L. fruit (Fig. 1a) juice was highly effective, economical, eco-friendly, and fast. The change in color of the precursor solution from colorless or pale yellow to blackish or brown following the addition of 1 mM AgNO_3_ aqueous solution implies the fabrication of Cm-AgNPs (as shown in inset Figs. 1A–B). The UV–Vis spectral studies were conducted to validate the Cm-AgNPs formation by *C. medica* L. juice. The absorbance peak in the UV–Vis spectroscopic analysis for the optical density (O.D.) was noted as a broad and asymmetric peak with a redshift at 487 nm equivalent to the surface plasmon resonance (SPR) (Fig. 1b).

**Fig. 2:**
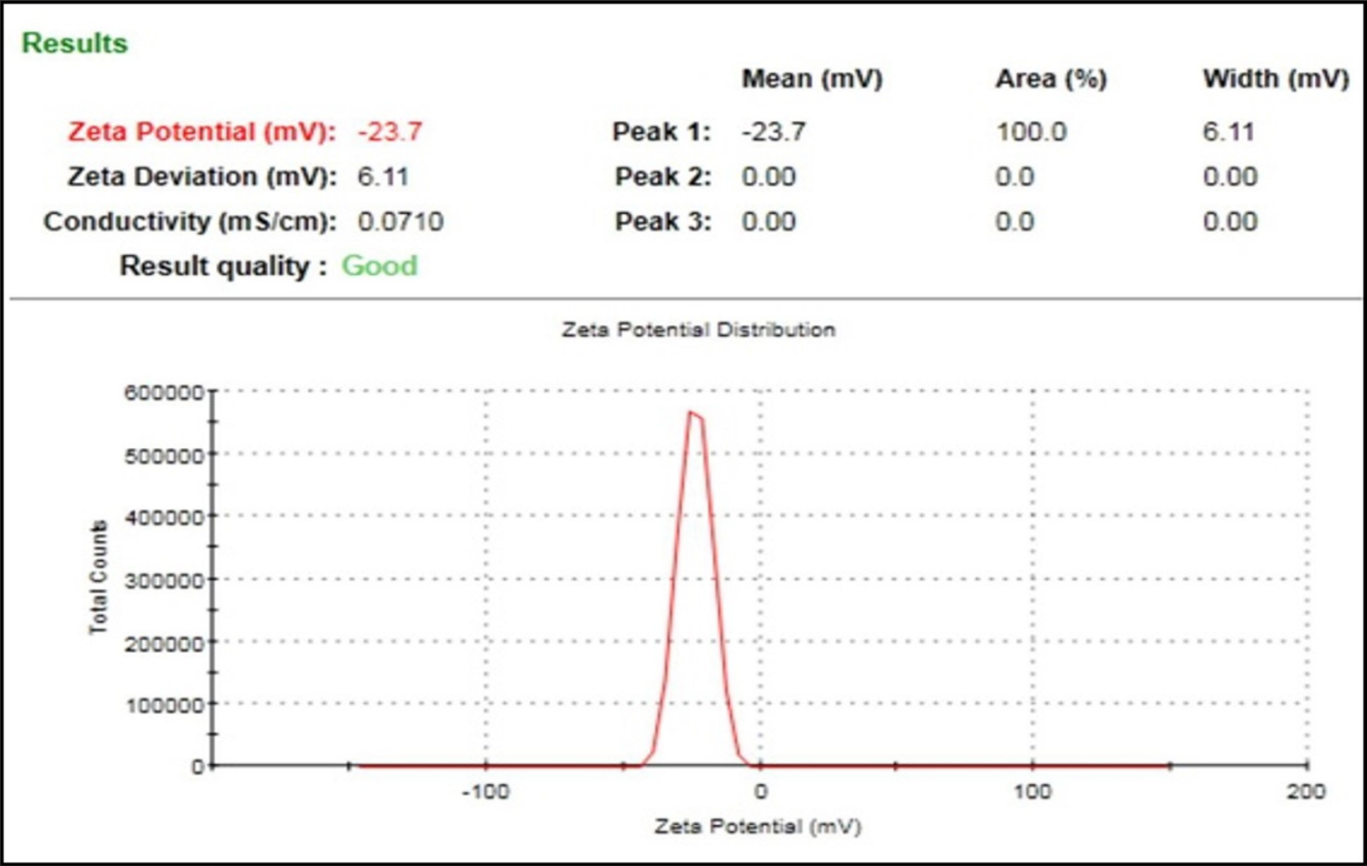
Zeta potential measurement of Cm-AgNPs originated to be − 23.7 mV

To understand the stability of Cm-AgNPs, the zeta potential was measured and detected as a negative value of − 23.7 mV (Fig. 2), which may be because of the capping of charged biomolecules.

**Fig. 3:**
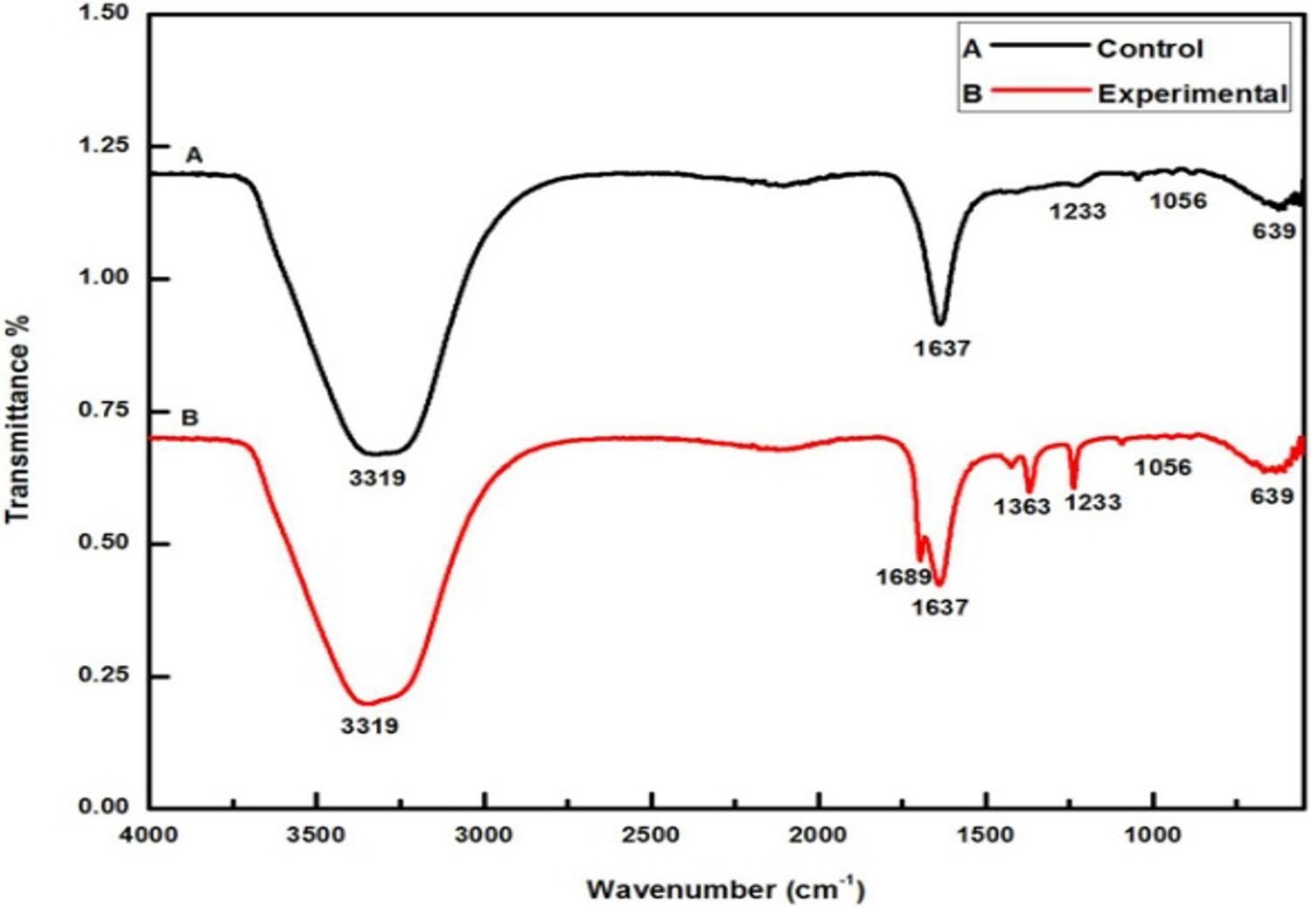
FTIR peaks of control and experimental samples for Cm-AgNPs

Moreover, the Cm-AgNPs were characterized by FT-IR spectral analysis to prove the manifestation of biomolecules on the synthesized Cm-AgNPs, which are believed to play a significant role in their reduction, capping, and stabilization [47]. Figure 3 provides evidence that in the Cm-AgNPs sample, bands shifted as compared to their corresponding controls. The predominant cause of band shifting is primarily attributed to the stretching vibration occurring within specific functional groups present in the biomolecules of plants, such as C=C vibration, C–O–C bond stretch vibration, and amide stretch, at 3319 cm^−1^, 1689 cm^−1^, 1637 cm^−1^, 1363 cm^−1^, 1233 cm^−1^, 1056 cm^−1^, and 639 cm^−1^, in agreement with their respective functional groups as presented in Table 1.

**Table 1:** Showing the experimental bands with their functional group assignment that confirms the acidic groups capping the Cm-AgNPs from citron juice

Experimental bands (Wavenumber cm^−1^)	Functional group assignment
3319	Phenols, Hydrogen bonded Alcohols
1689	Aldehydes, Carboxylic acids, Esters, Ketones
1637	Alkenes
1363	Alkanes
1233, 1056	Alcohols, Carboxylic acids, Esters, Ethers
639	Aliphatic bromo compounds

**Fig. 4:**
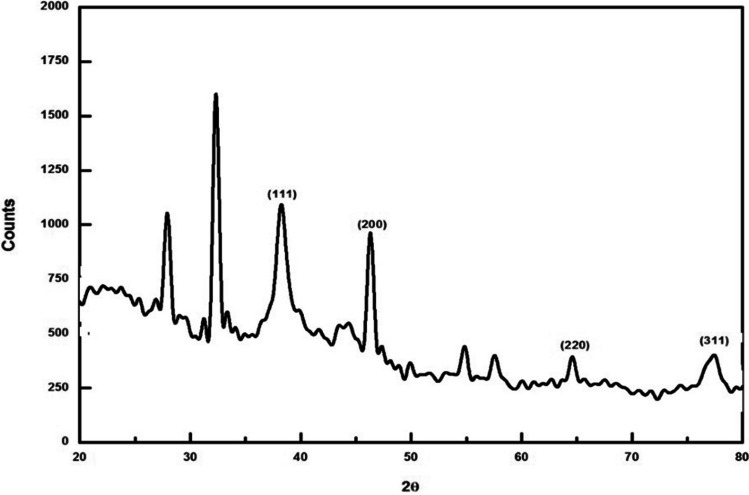
XRD analysis of Cm-AgNPs

Further, these Cm-AgNPs subjected to the X-ray Diffraction (XRD) pattern study displayed the peaks at the positions of 38°, 44°, 66°, and 78° of 2θ angles, showing the incidence of Miller indices (111), (200), (220), and (311) corresponding to the facets of the face-centered cubic “*fcc*” nature of crystalline silver, which confirms the synthesis of Cm-AgNPs (JCPDS file no. 04-0783). The “*fcc*” crystalline structure of Ag proves the fabrication of AgNPs (Fig. 4). In line with the earlier literature, the additional peaks in XRD were because of the bio-organic phase mount on the surface of AgNPs, and the greater thickness of the peaks affects their smaller size [39].

**Fig. 5:**
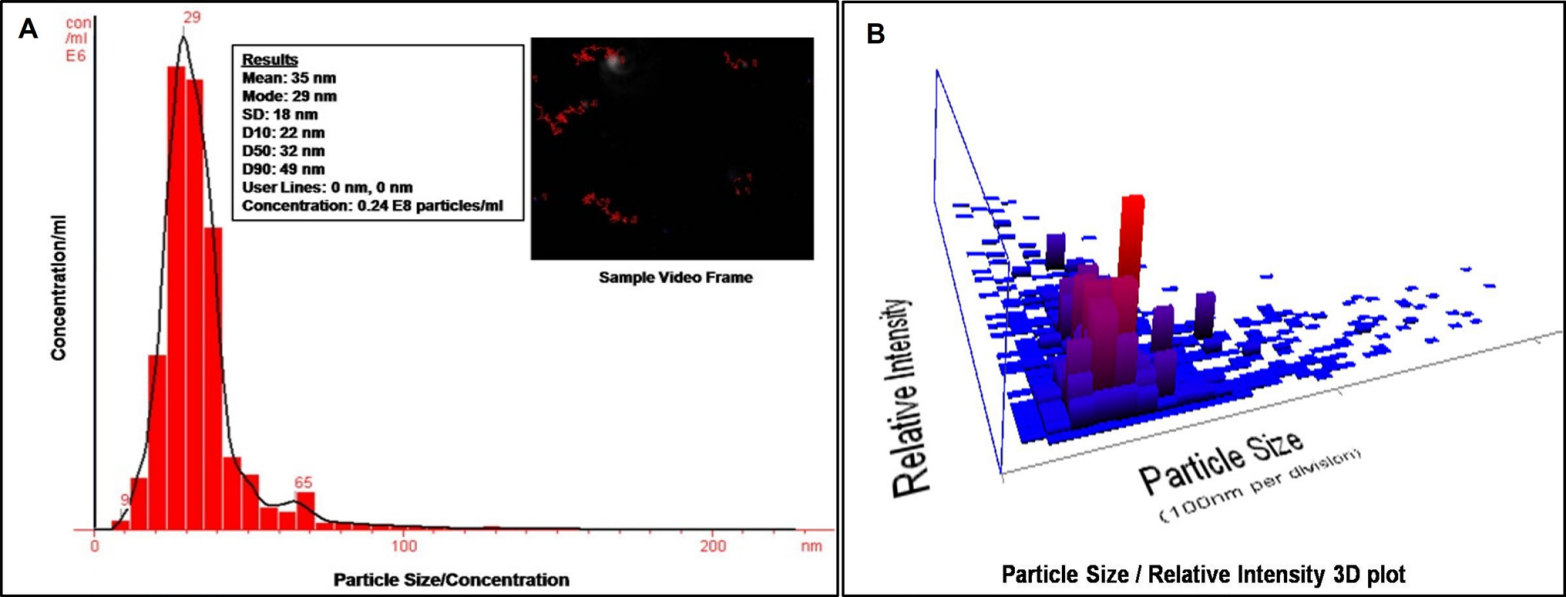
Nanoparticles Tracking and analysis (NTA) of Cm-AgNPs **A** The experimental results the mean particles size was 35 nm with the concentration of 2.4 × 10^8^ particles mL^−1^ **B** 3D plot for relative intensity view

The Cm-AgNPs were then characterized using NanoSight LM20 by a specific software program, NTA. The NTA system observes individual NPs and ranges them based on a particle-by-particle basis. Fundamental drawbacks in methods comparable to dynamic light scattering (DLS) or photon-correlation spectrophotometry that give a mean particle size get thwarted by NTA. It demonstrates a mean size of biofabricated Cm-AgNPs of 35 ± 18 nm with a concentration of 2.4 × 10^8^ particles mL^−1^ (Figs. 5A and B).

**Fig. 6:**
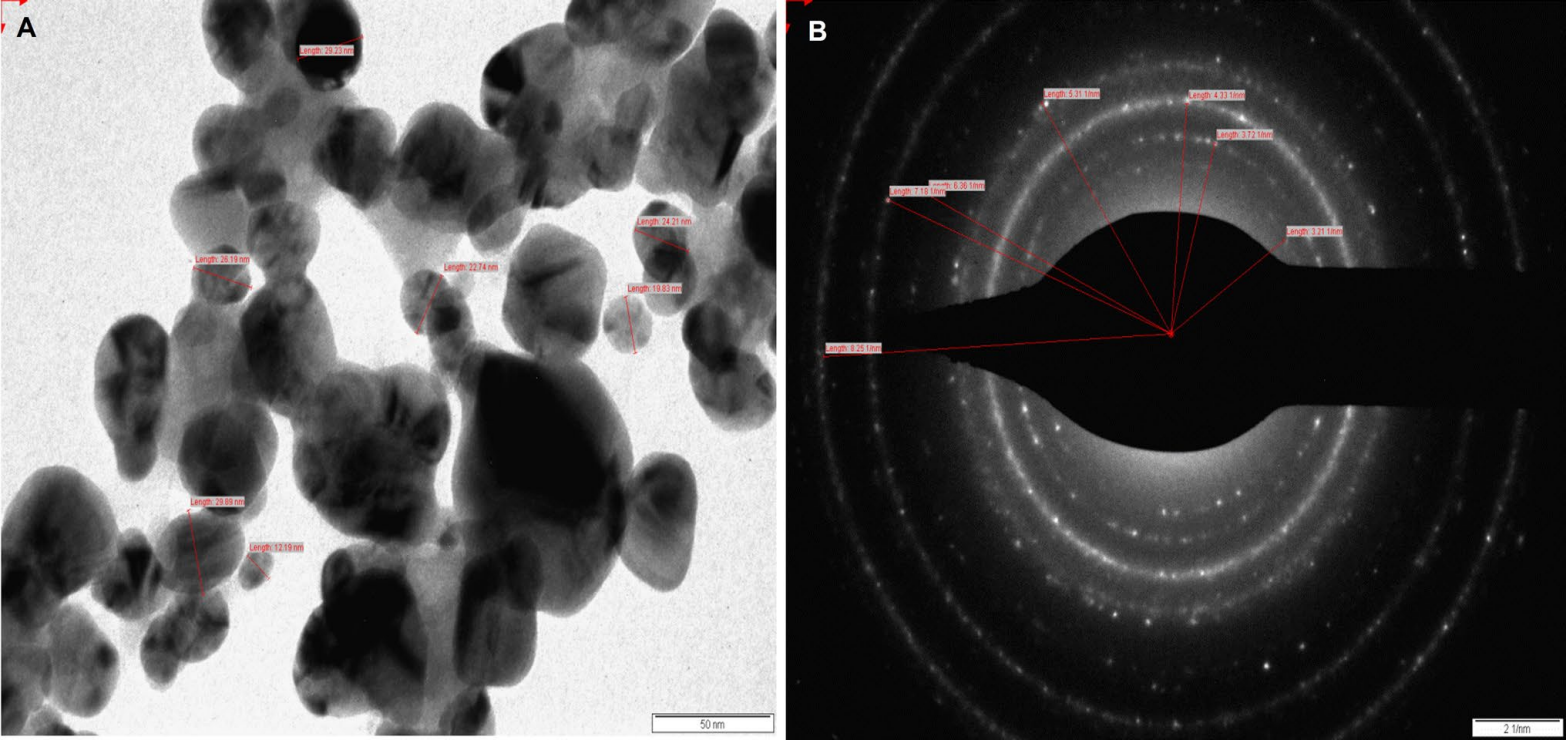
**A** TEM analysis showed the spherical Cm-AgNPs with a size in the range of 20–60 nm **B** SAED pattern showing crystal rings

In addition to XRD analysis, the fabrication of Cm-AgNPs was determined by TEM and SAED pattern studies to achieve the desired topology and size, which is impossible with NTA. The TEM micrograph showed that the majority of the Cm-AgNPs are polydisperse and spherical, but some larger, more noticeable NPs with irregular shapes were also visible. Consequently, a rise in polydispersity was observed in the 20–60 nm range, with a mean of 24 ± 3 nm (Fig. 6A). The SAED pattern exhibited crystal rings comparable to the “face-centered cubic (*fcc*)” crystal structure of synthesized Cm-AgNPs (Fig. 6B).

**Fig. 7:**
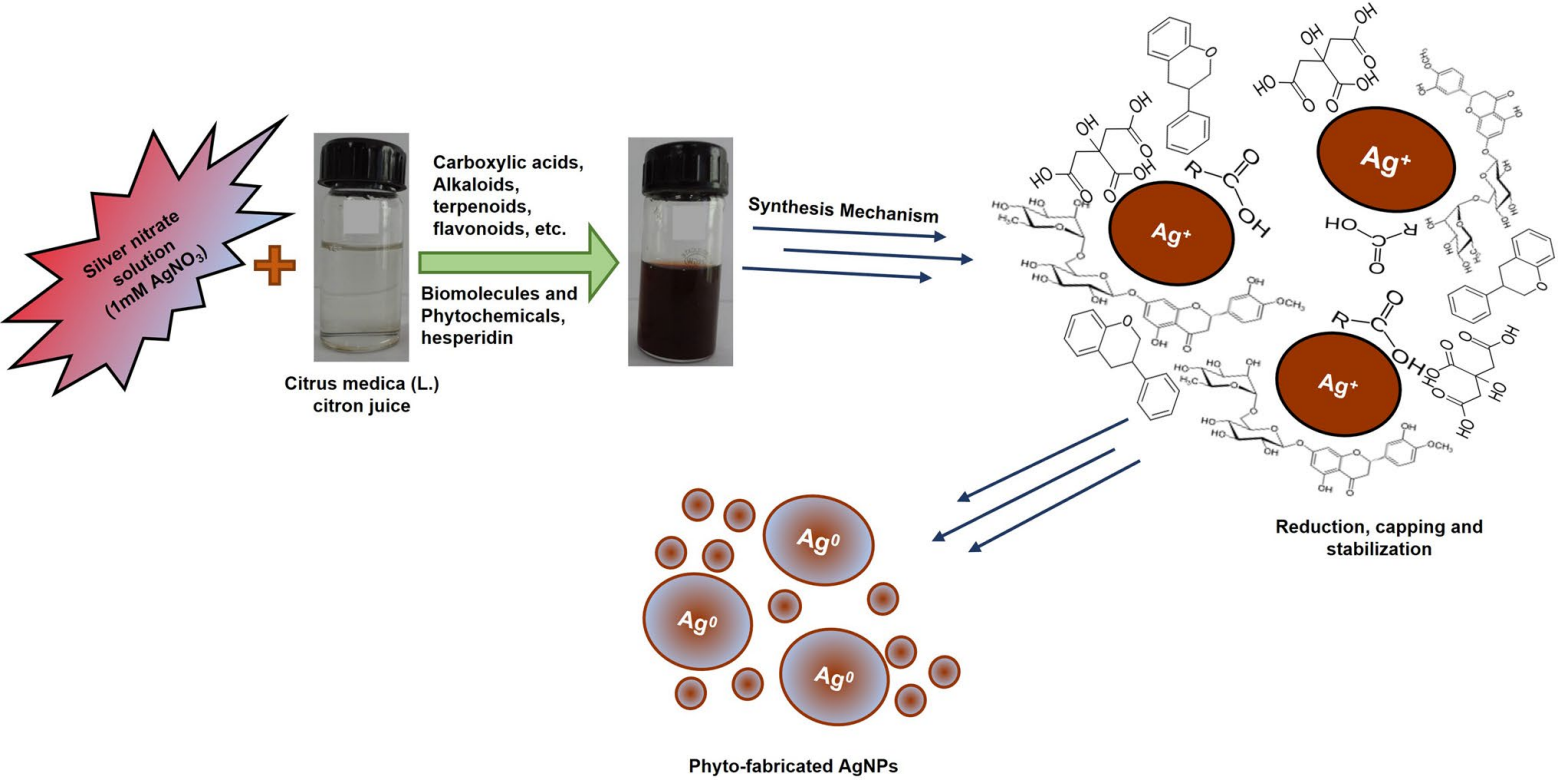
Hypothetical Mechanism involved in Biofabrication of Silver Nanoparticles (Cm-AgNPs) from Citron juice

Throughout the fabrication of AgNPs by the green method, the most essential objective is to improve reliability and security, thus preventing the economic and ecological destruction of threatening natural resources. Temperature, reaction pH, manufacturing time, equivalent concentrations of metal ions, and plant extract can all have a substantial impact on the quality, origin, yield, and characteristics of the resulting AgNPs [21]. Although a wide range of plants and their components may include a variety of biomolecules that can serve as reducing, capping, and stabilizing agents throughout the fabrication process, the choice of certain plants and plant parts becomes essential for efficient phytosynthesis. The biomolecules from plants might also impact the properties of AgNP surfaces. The hypothetical mechanism involved in biofabrication of Cm-AgNPs using citron juice is illustrated in Fig. 7.

The fabrication of nanoparticles from live resources is considerably safer and more sound than physical and chemical approaches. A biological method using plants to fabricate stable AgNPs appears to be advantageous over the other techniques. Other biomolecules employed in the fabrication of AgNPs involve biopolymers (like alginate, chitosan, cellulose, lignin, and polypeptides), amino acids, enzymes, and starch. It is assumed that the biomolecules from plants include mainly secondary metabolites, for example, amino acids, alcoholic compounds, alkynes, enzymes, proteins, polysaccharides, vitamins, terpenoids, alcohols, tannins, carotenes, sugars, and many more. These may reduce the Ag^+^ ions to Ag^0^ [22].

The main substances that could be implicated in the fabrication of AgNPs in citron juice are flavonoids (such as hesperidin, hesperetin, rutin, etc.), ascorbic acid, phenolic compounds, and various carboxylic acids as shown in Fig. 7. It is hypothesized that the carboxyl (-COOH) group found in glutamine acid and aspartic acid, as well as the -OH group in tyrosine, are functional groups that stabilize silver ions to produce nanoscale polydisperse AgNPs. Besides this, the hydroxyl and carbonyl groups of flavonoids play a vital role in reducing Ag ions by ion chelation through the flavonoids. Three primary flavonoids function as reducing, capping, or stabilizing agents for the Ag^+^ to Ag^0^ reduction: flavon-3,4 ol and flavon-3,4 diol [13, 50].

Even though nearly all of the earlier research suggested that the biomolecules participated in a process that integrated simultaneous reduction and capping, none of them could identify the phyto-biomolecules responsible for each function. Still, most of the literature on the green fabrication of AgNPs concentrates exclusively on application efficiency. Only some address the possible limitations and challenges of this method. It is essential to ascertain the perfect phytochemicals contributing to the biofabrication of AgNPs to elucidate the mechanism. However, a significant obstacle to understanding the connections between morphology, production, and compounds in plant extract is a lacuna of proper plant extract characterization based on common phytoconstituents [35, 43].

### Antifungal activity of biofabricated Cm-AgNPs

**Fig. 8:**
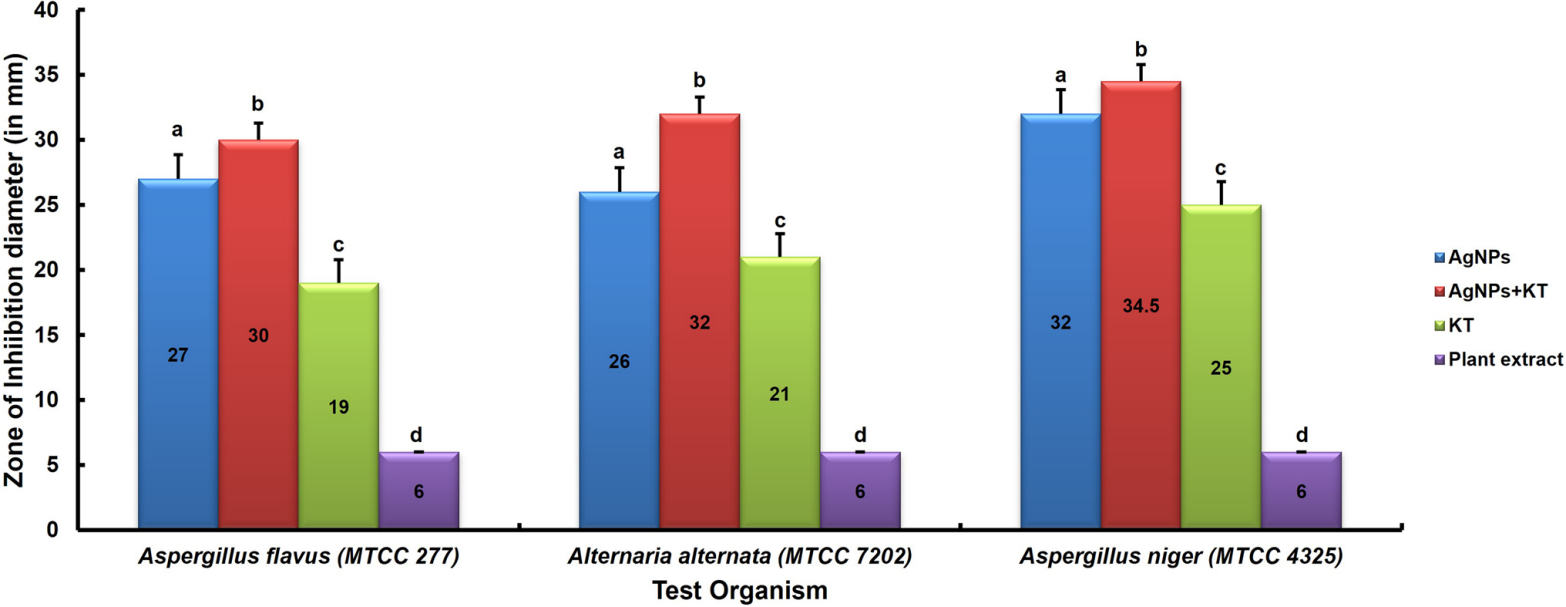
Antifungal activity of Biofabricated Silver Nanoparticles (Cm-AgNPs) against **A** *Aspergillus flavus* (MTCC 277), **B** *Alternaria alternata* (MTCC 7202), **C** *Aspergillus niger* (MTCC 4325) [where (**a**) AgNPs, (**b**) AgNPs + Ketoconazole (KT), (**c**) Ketoconazole (KT) alone, (**d**) plant extract]

It has been observed that the antifungal activity evaluation by the Kirby-Bauer disk diffusion method exhibited considerable activity against the tested crop pathogens, in which the letters indicated on the graph are for treatments such as (a) for Cm-AgNPs, (b) for Cm-AgNPs in combination with ketoconazole (KT), (c) for ketoconazole (KT) alone, and (d) for plant extract (Fig. 8). By NTA analysis, it has been observed that the concentration of Cm-AgNPs was 2.4 × 10^8^ particles mL^−1^, which was directly used during the evaluation of antifungal activity (20 µL/disk). The Cm-AgNPs alone and combined with the antifungal agent ketoconazole (KT) showed significant activity, as revealed by inhibition zones (ZOI) diameter calculations in mm. The study demonstrated that the optimal ZOI value after treatment of biofabricated Cm-AgNPs was 32 mm for *Aspergillus niger* (MTCC 4325) > 27 mm for *A. flavus* (MTCC 277) > 26 mm for *Alternaria alternata* (MTCC 7202). The ZOI was enhanced when biofabricated Cm-AgNPs and ketoconazole (KT) were used in combination. They showed a synergistic effect on the tested pathogens. The ZOI was found to be maximum in *A. niger* (34.5 mm), minimum in *A. flavus* (30 mm), and in between for *A. alternata* (32 mm). The control (plant extract) did not show any activity on these pathogens. The experiment was carried out in triplicate.

## Discussion

The Cm-AgNPs showed remarkable antifungal efficacy against the tested post-harvest crop pathogenic fungi, viz., *A. niger*, *A. flavus*, and *A. alternata*. The capping phenomenon preceded the stabilization of the biofabricated Cm-AgNPs and reduced their aggregation. Earlier, it was also indicated that AgNPs are the most effective agents for combating infections and diseases [44, 60]. The citron juice component effectively reduced silver ions (Ag^+^) to AgNPs (Ag^0^). This was verified through UV–Vis absorption spectroscopy, evident from the presence of their SPR peak within the 410–440 nm range [30, 57]. This established the silver ion’s reduction [6, 18]. Upon reduction of pure Ag^+^ to Ag^0^ with the fruit juice, SPR (487 nm) and peak broadening revealed that the poly-dispersed particles were formed, which is a primary leading and substantial indication for the fabrication of Cm-AgNPs. They prompted the color shift observed during this analysis. The redox reaction took place during the synthesis of AgNPs. Nevertheless, the reflection of this peak was designated to the SPR of AgNPs. The fruit juice could form a complex with AgNPs, which may stabilize the system and counteract the agglomeration of AgNPs. The silver ions can reduce their neutral silver atoms by using a biological source like algae, bacteria, fungi, or plant extracts as a reducing or oxidizing agent. They can then grow and form an aggregate, stabilizing and capping silver ions to form AgNPs. It was noted that the biosynthesized Cm-AgNPs are mostly spherical [33]. The current findings corroborate the previous studies that reported color change in the filtrate was caused by the excitation in SPR vibration of Cm-AgNPs. Recently, Alshameri et al. [6] reported the phytofabrication of AgNPs using *Rumex nervosus* leaf extract (Rn-AgNPs), which showed the SPR at 450 nm for synthesized Rn-AgNPs. From the FTIR spectral analysis, the role of *Citrus medica* fruit juice in the biosynthesis of Cm-AgNPs was validated according to the peak shift detected for the functional group assignment as given in Table 1.

Cm-AgNPs are structurally and morphologically depicted for shape, particle size, and nature by NTA, XRD, TEM, and SAED pattern analysis. The XRD analysis demonstrated the Miller indices were analogous to the facets of the “*fcc*” crystalline structure of silver, confirming the production of Cm-AgNPs. The Cm-AgNPs’ crystalline structure was manifest in XRD analysis and was identical to the other biosynthesized AgNPs [10, 17, 46]. It indicates the crystallization of the surface bio-organic phase of the AgNPs [10, 56]. Nanoparticles Tracking and Analysis (NTA) revealed synthesized Cm-AgNPs of 35 ± 18 nm with a concentration of 2.4 × 10^8^ particles mL^−1^, which corresponds to the DLS studies of Rn-AgNPs that showed the mean size of biosynthesized AgNPs was 62 ± 3 nm [6].

Based on the TEM and SAED patterns, biofabricated Cm-AgNPs ranged from 20 to 60 nm, with an average size of 24 ± 3 nm, corresponding to the results reported by Chandrasekharan et al. [17]. The TEM analysis of AgNPs synthesized from *Gmelina arborea* extract exhibited spherical and distinct AgNPs with a diameter of 34–40 nm. The zeta potential analysis was made to ascertain the charge value on the Cm-AgNPs surface. The stability of biofabricated Cm-AgNPs was observed to be − 23.7 mV, corresponding to the zeta potential value measured for Rn-AgNPs (− 22 mV) in the study by Alshameri et al. [6]. It has been suggested that the higher the zeta potential, the more stable the NPs in their colloidal suspension [42]. Cm-AgNPs showed the main antifungal activity against all the post-harvest crop pathogens tested. The diameter of inhibition zones (ZOI) after the treatment was 32 mm against *A. niger*, 27 mm against *A. flavus*, and 26 mm against *A. alternata*. The activity improved when biofabricated Cm-AgNPs were combined with ketoconazole (KT). It showed a synergistic effect on the tested pathogens, which were found to exhibit a maximum ZOI of 34.5 mm against *A. niger*, a minimum of 30 mm against *A. flavus*, and a maximum of 32 mm against *A. alternata*. There are several reports on AgNP’s antimicrobial efficacy against pathogens causing diseases in plants [5, 19, 20, 40]. The data of this study correspond with the earlier published data that observed the significant antifungal activity of biogenically synthesized AgNPs by *Amaranthus retroflexus* leaf extract against *Macrophomina phaseolina*, *A. alternata*, *Fusarium oxysporum* [4, 7], against *Alternaria porri* [19], *Rhizoctonia solani* [20],and *Colletotrichum truncatum* [29]. The antifungal activity demonstrated by AgNPs against post-harvest crop pathogens opens the door towards developing novel antifungal agents using biogenically synthesized Cm-AgNPs for sustainable agriculture.

## Conclusions and future perspectives

The fabrication of green Cm-AgNPs involves an eco-friendly method utilizing *Citrus medica (*L.) fruit (citron) juice. This synthesis was conducted at room temperature, devoid of harmful chemicals and high temperatures, making it a lucrative process. Citron fruits are readily available in forests as well as in medicinal plant gardens; hence, the process is economical. Biofabricated Cm-AgNPs were polydisperse, crystalline, spherical, and differentially sized (20–60 nm). The underlying bioactivity of fruit juice was demonstrated in elevated quantities in various Cm-AgNPs. Together with Cm-AgNPs-KT, significant fungal inhibition was recorded against the tested post-harvest fungal pathogens, indicating that Cm-AgNPs is a suitable candidate for the development of further broad-spectrum antifungal agents for agricultural crop protection. At a concentration of 20 uL/disk of Cm-AgNPs, it showed more significant antifungal activity than that of KT, so it could be a cheaper alternative compared to that of standard antifungals. These non-toxic, biofabricated Cm-AgNPs have excellent antifungal efficacy against post-harvest fungal crop pathogens, viz., *A. niger* (MTCC 4325), *A. flavus* (MTCC 277), and *A. alternata* (MTCC 7202); therefore, they could be employed for their management of crop diseases. The current research concludes that our biofabrication of AgNPs technique is relatively easy, cost-effective, fast, reproducible, stable, eco-friendly, and acquiescent in scaling up for the large-scale production of AgNPs in a sustainable manner. This innovative approach may be further improved for exploring sustainable management of crop diseases. By employing Cm-AgNPs, nanoscale products can be developed to combat post-harvest pathogenic fungi in crops, which will ultimately benefit society and contribute towards achieving the SDGs ‘#Zero Hunger’ goal.

## Data Availability

All data related to this research has been included in the article.
